# Supplementary material for: SETD8 inhibition targets cancer cells with increased rates of ribosome biogenesis
Source: Cell Death Dis. 2024 Sep 28;15(9):694. doi: 10.1038/s41419-024-07106-6 (PMC11438997; doi:10.1038/s41419-024-07106-6)
Supplement: Supplementary file 1 — Supplementary Figures and Tables [file 41419_2024_7106_MOESM1_ESM.pdf]

Murga et al **FigS1**

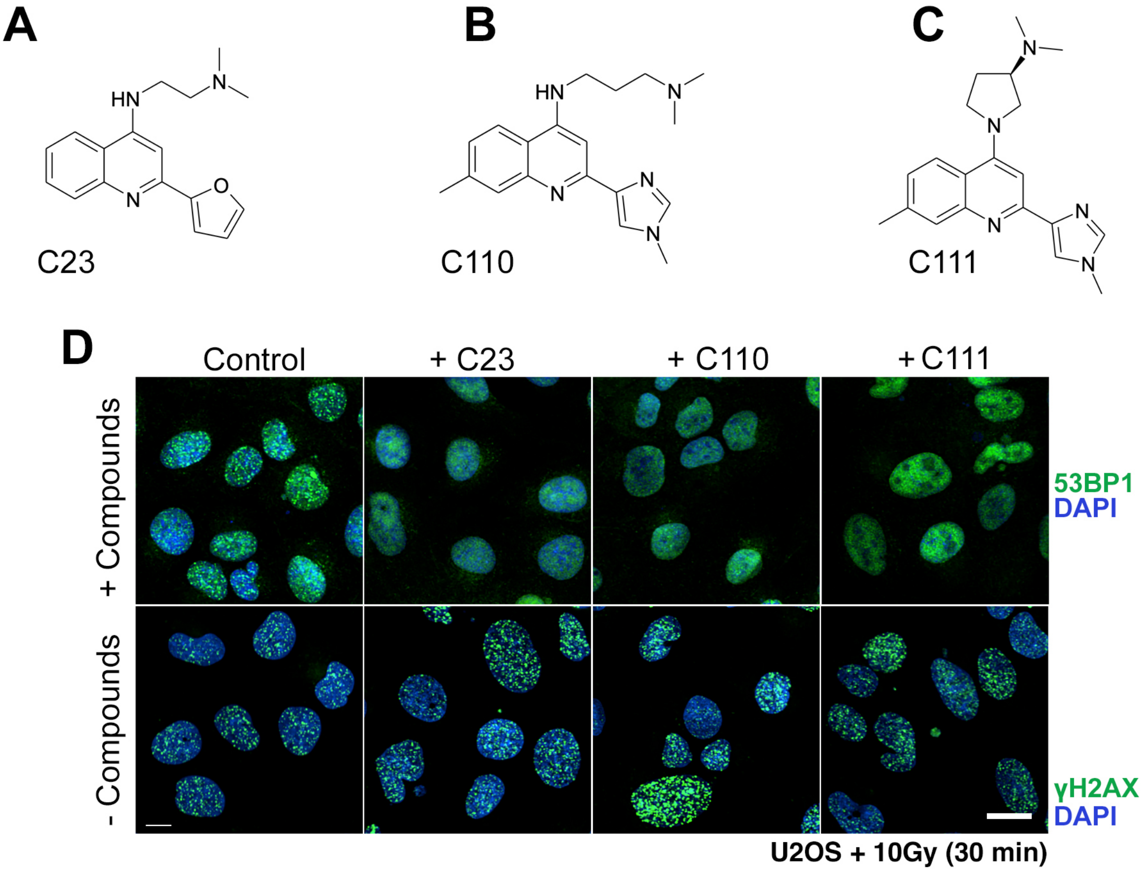

Figure S1. C23 analogs and their effect on 53BP1 foci formation. (**A-C**) Chemical structures of C23 and 2 analogs that are also efficient in inhibiting 53BP1 foci formation. (**D**) Immunofluorescence of 53BP1 (green) in U2OS cells, treated or not with C23, C110 or C111 (10 μM), 45 min after exposure to 10Gy of IR. Scale bar (white) indicates 5 μm.

Murga et al **FigS2**

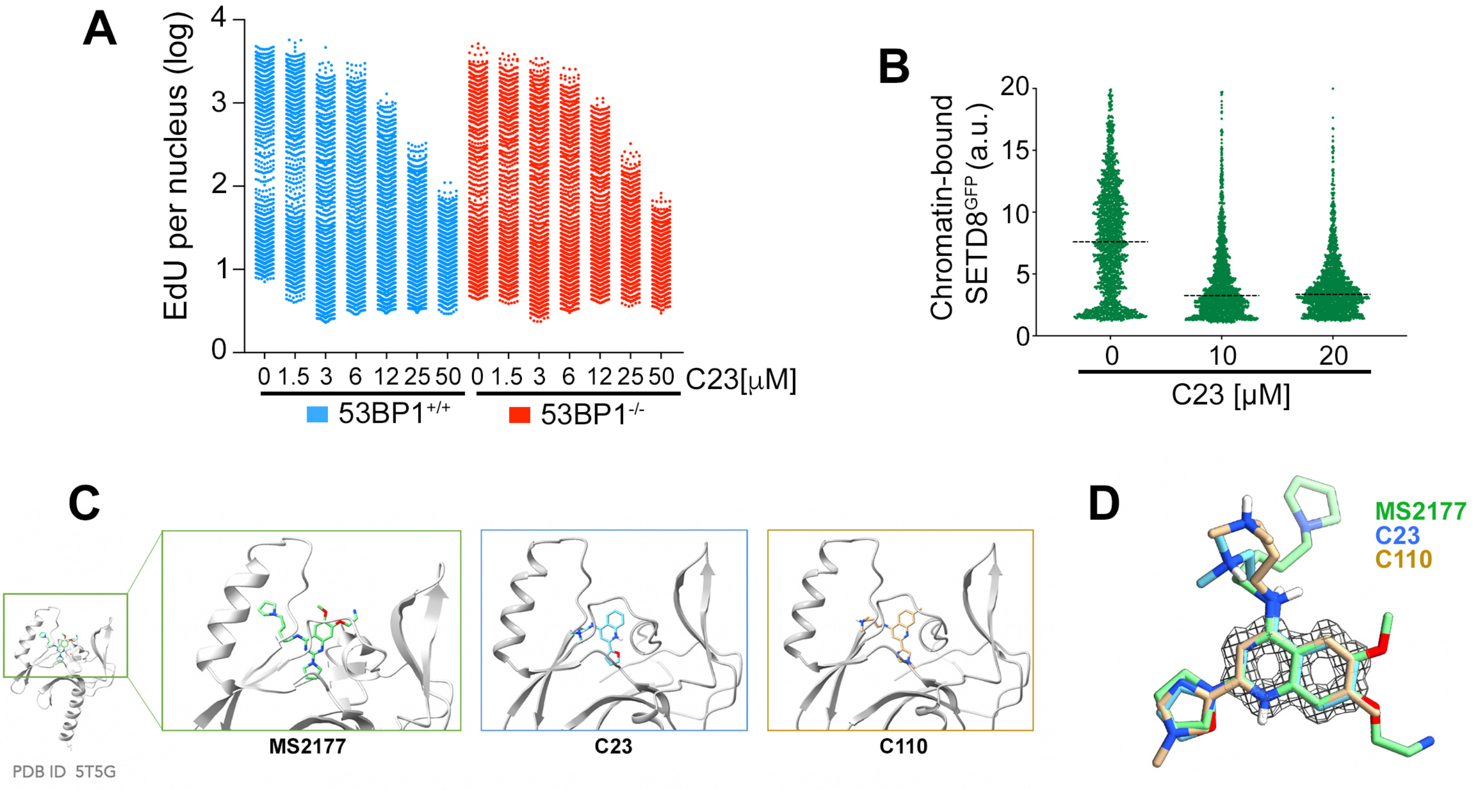

Figure S2. 53BP1-independent functions of C23 relate to SETD8 inhibition. (**A**) HTM-dependent quantification of EdU levels per nucleus, in *53BP1*$^{+/+}$ and *53BP1*$^{-/-}$ MEF treated with increasing concentrations of C23 for 45 min. (**B**) HTM-dependent quantification of the nuclear levels of chromatin-bound SETD8^{GFP} in U2OS cells exposed for 45 min to increasing concentrations of C23. A detergent-based protocol that extracts the nuclear-soluble fraction of proteins that are not bound to chromatin was applied previous to image acquisition. (**C**) Docking of C23, C110 on the catalytic domain of SETD8, using as a reference the previously published structure of MS2177-bound SETD8 (PDB 5T5G). (**D**) Alignment of the chemical structures of MS2177, C23 and C110.

Murga et al **FigS3**

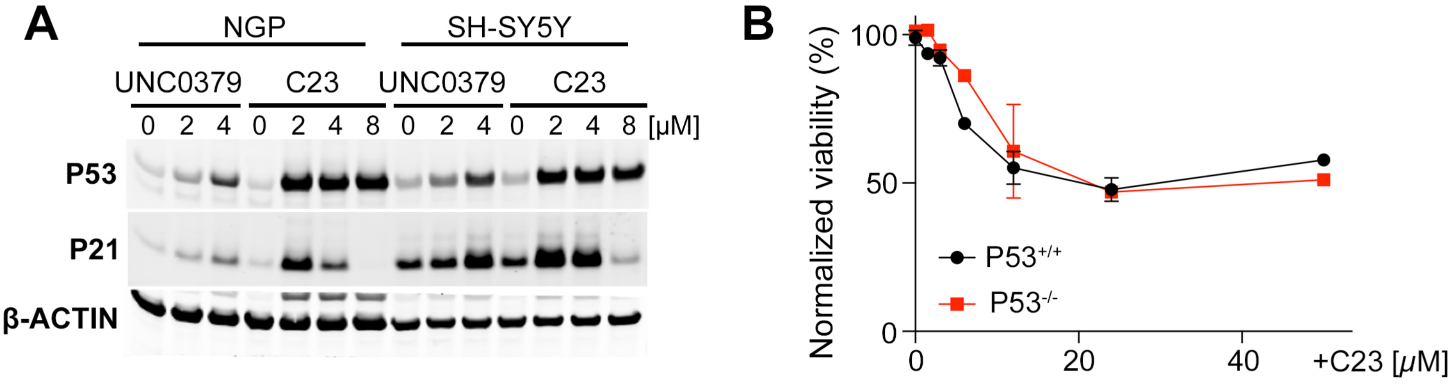

Figure S3. C23 toxicity is p53-independent SETD8 inhibition. (**A**) WB illustrating the levels of P53 and P21 in independent neuroblastoma cell lines treated with increasing concentrations of C23 for 8 h. β-ACTIN levels are shown as a loading control. (**B**) Effect of increasing concentrations of C23 on the viability of $P53^{+/+}$ and $P53^{-/-}$ MEF.

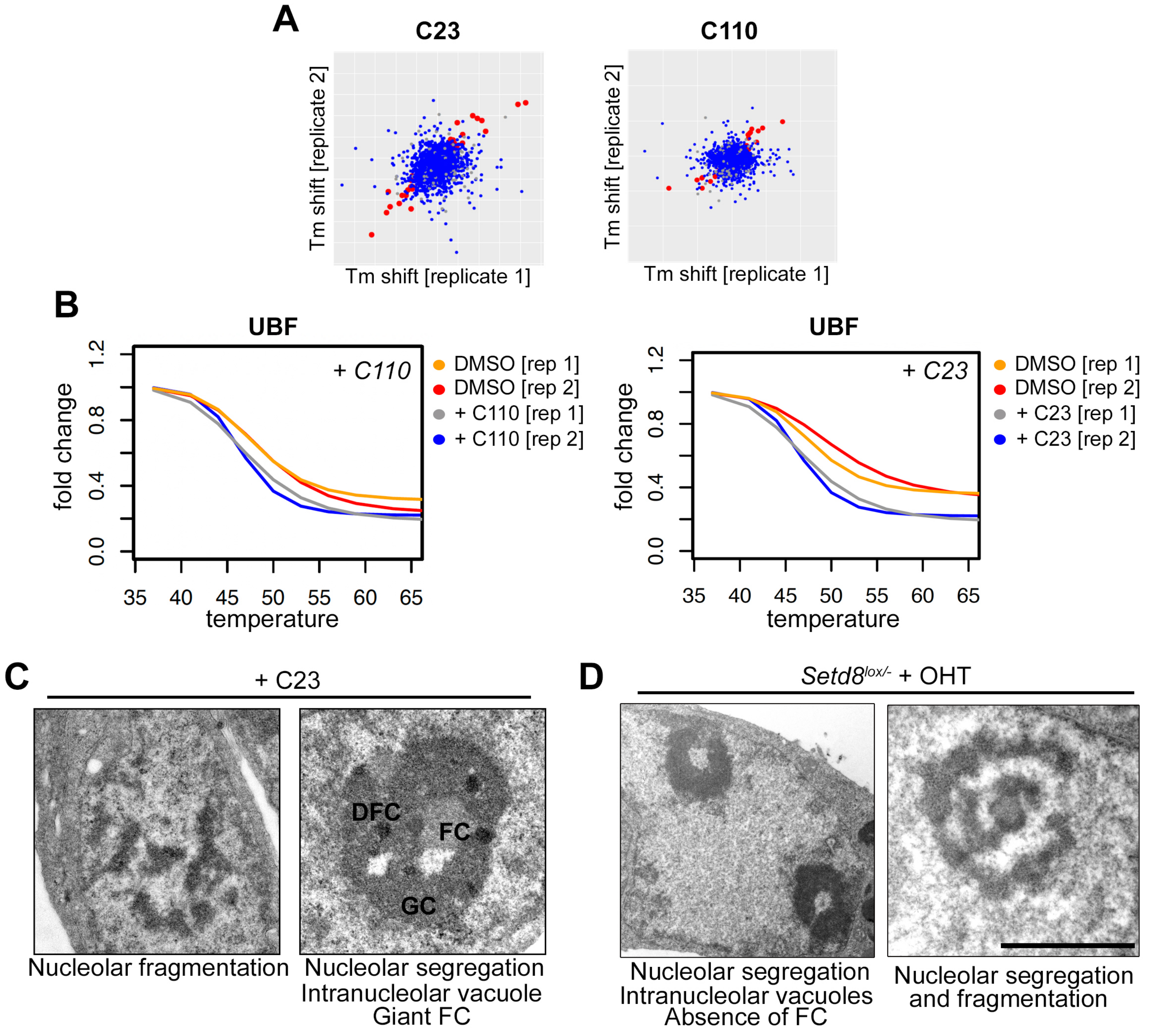
A
C23
C110
Tm shift [replicate 2]
Tm shift [replicate 1]
B
UBF
+ C110
+ C23
fold change
temperature
0.0
0.4
0.8
1.2
35
40
45
50
55
60
65
DMSO [rep 1]
DMSO [rep 2]
+ C110 [rep 1]
+ C110 [rep 2]
+ C23 [rep 1]
+ C23 [rep 2]
C
+ C23
DFC
FC
GC
Nucleolar fragmentation
Nucleolar segregation
Intranucleolar vacuole
Giant FC
D
Setd8lox/- + OHT
Nucleolar segregation
Intranucleolar vacuoles
Absence of FC
Nucleolar segregation
and fragmentation

Figure S4. Targeting SETD8 generates nucleolar stress. (**A**) Overall correlation of melting temperature (Tm) shifts between the two biological replicates of the TPP experiment. (**B**) Impact of increasing the temperature on UBF levels as assessed by TPP in U2OS cells treated with C23 or C110 for 25 min. Data from 2 biological replicates is shown. (**C, D**) Representative TEM images from U2OS cells treated with C23 (25 μM) for 45 min (**C**) or *Setd8*$^{+/-}$ and *Setd8*$^{lox/-}$ mESC 24 h after exposure to 4-OHT (1 μM). Scale bar represents 2 μM.

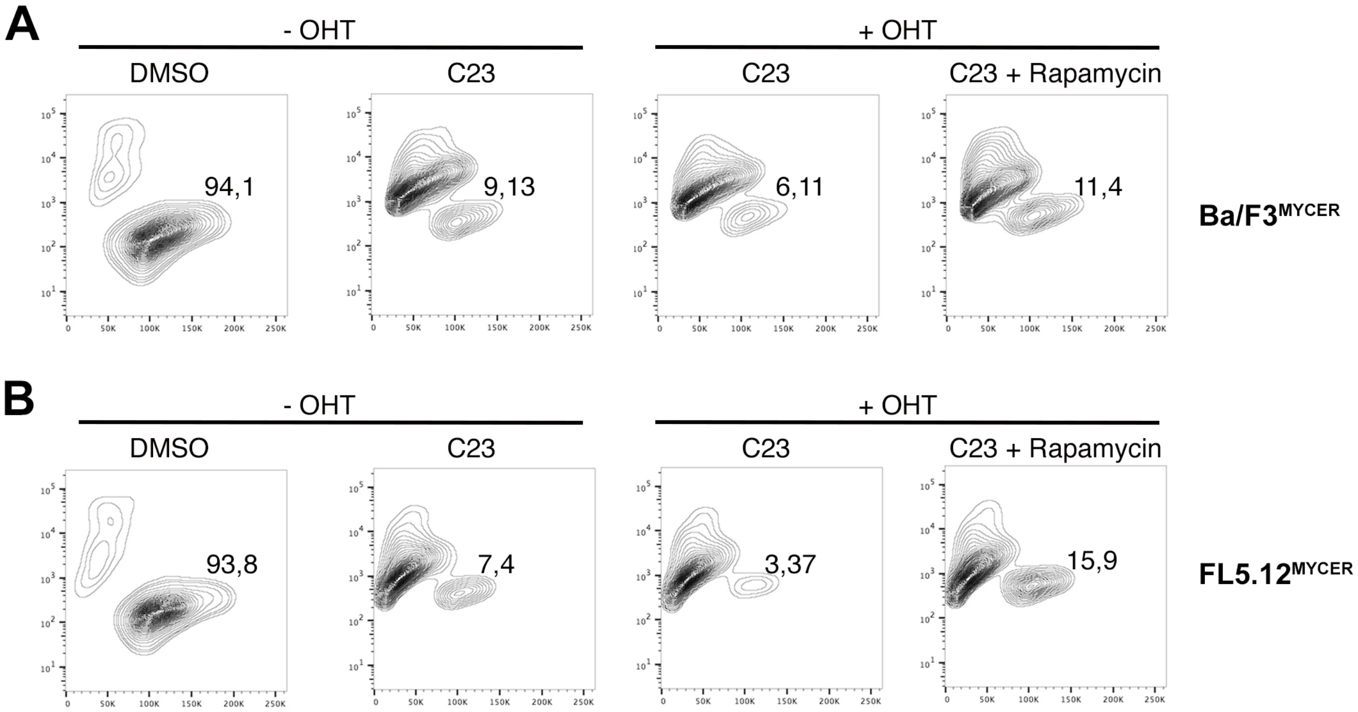
A
- OHT
+ OHT
DMSO
C23
C23
C23 + Rapamycin
94,1
9,13
6,11
11,4
Ba/F3^MYCER
B
- OHT
+ OHT
DMSO
C23
C23
C23 + Rapamycin
93,8
7,4
3,37
15,9
FL5.12^MYCER

Figure S5. C23 toxicity correlates with MYC and mTOR activity. (**A, B**) Flow cytometry profiles (*Y*-axis: DAPI; *X*-axis: FSC) of Ba/F3^{MYCER} and FL5.12^{MYCER} cells treated with OHT (1 mM, 24 h) in the presence or absence of C23 (10 μM) or Rapamycin (50 nM). The percentage of viable cells is indicated in each profile.

Murga et al **Original western blots**

Fig. 2B

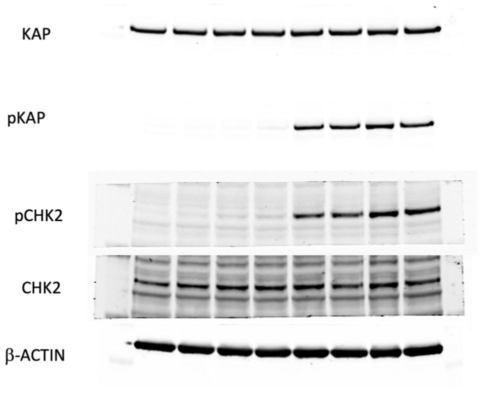

Fig. 4A

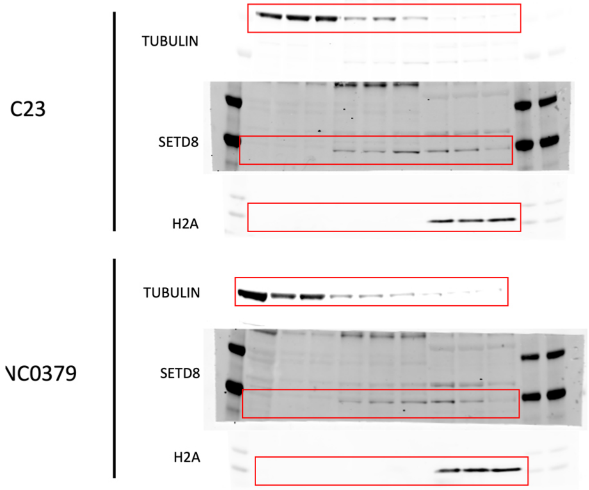

Fig. 4B

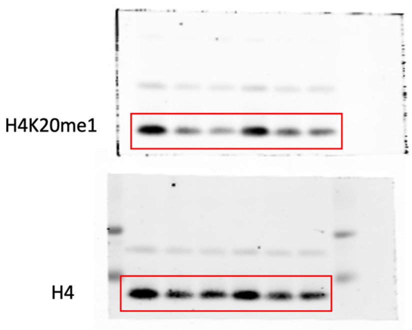

Fig. 6B

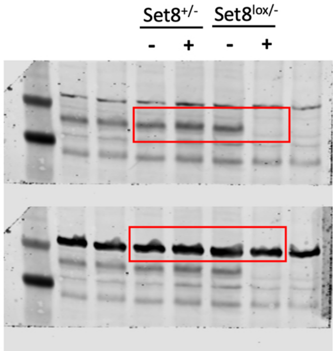

Fig. S3A

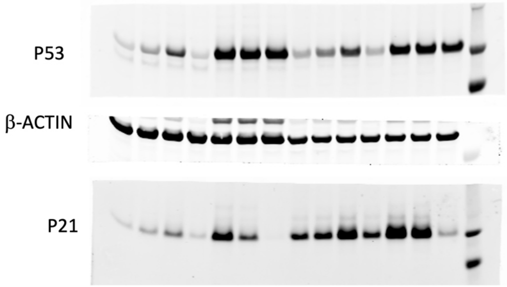

TABLE S1. List of original hits selected from the virtual screen.		
**COMPOUND**	**NUMBER**	**NAME**
AW00847	1	4-(4-fluorobenzyl)-N-phenyl-1,4-diazepane-1-carboxamide
RH01524	2	N-((4,4-dimehtyl-2,6-dioxocyclohexylidene)methyl)aspartic acid
SEW05754	3	3-{[2-(4-methylpiperazino)phenyl]methylene}-1H-indol-2-one
RDR02556	4	N1-(2,6-dimethylphenyl)-3-piperidinopropanamide
KS-5103	5	(-)-N-(((S)-1-Ethyl-2-pyrrolidinyl)methyl)-5-sulfamoyl-o-anisamide
E101	6	S-(−)-3-Chloro-5-ethyl-N-[(1-ethyl-2-pyrrolidinyl)methyl]-6-hydroxy-2-methoxybenzamide hydrochlo
M3189	7	N-(2,6-Dimethylphenyl)-1-methyl-2-piperidinecarboxamide hydrochloride
STK134574	8	1-(diethylamino)-3-[(1,1-dioxido-1,2-benzothiazol-3-yl)amino]propan-2-ol
STL059340	9	3-hydroxy-N,N,3-trimethyl-4-phenylbutan-1-aminium
STK538232	10	1-azabicyclo[2.2.2]oct-3-yl(dithiophen-2-yl)methanol
STL356215	11	N-(3-chlorophenyl)-2-(pyrrolidin-1-yl)acetamide
STK379741	12	N-{4-[methyl(1-methylpiperidin-4-yl)sulfamoyl]phenyl}acetamide
STK887751	13	8-[(dimethylamino)methyl]-7-hydroxy-3-(1-methyl-1H-benzimidazol-2-yl)-2H-chromen-2-one
STL059340	14	3-hydroxy-N,N,3-trimethyl-4-phenylbutan-1-aminium
STK054778	15	2-{3-[3-(dimethylamino)propyl]-2-imino-2,3-dihydro-1H-benzimidazol-1-yl}-1-phenylethanol
STK540953	16	8-{[2-(dimethylamino)ethyl]amino}-7-ethyl-1,3-dimethyl-3,7-dihydro-1H-purine-2,6-dione
STL169956	17	N-(4-methoxyphenyl)-4-methylpiperazine-1-carbothioamide
AW01002	18	N-[2-(dimethylamino)ethyl]-5-(2-methyl-1,3-thiazol-4-yl)thiophene-2-sulfonamide
AG-205/15424896	19	N-[3-(dimethylsulfamoyl)phenyl]-4-oxo-3-phenyl-2-thioxo-1H-quinazoline-7-carboxamide
AK-968/36977036	20	(2R)-3-hydroxy-2-[(2-isopropyl-4-methyl-6-oxo-1H-pyrimidine-5-carbonyl)amino]propanoic
PH004881ALDRICH	21	4-(dimethylamino)-2,2-diphenylpentanamide
S561274	22	4-amino-alpha-diethylamino-O-cresol
SML2187	23	N2-[2-(2-Furanyl)-4-quinolinyl]-N1,N1-dimethyl-1,2-ethanediamine
CDS020251	24	2-[(4-Methylpiperazin-1-yl)carbonyl]aniline

TABLE S2. List of reagents used in this study.

REAGENT or RESOURCE	SOURCE	IDENTIFIER
Antibodies		
Rabbit anti-53BP1	Novus	Cat#100-304A2
Mouse anti-H2AX	EMD Millipore	Cat#05-636
Mouse anti-p53	Santa Cruz	Cat#sc-6243
Mouse Anti-KAP	BD Pharmingen	Cat#610680
Rabbit anti-FBL	Cell Signaling	Cat#2639
Mouse anti-UBF	Santa Cruz	Cat#sc-13125
Rabbit anti-NCL	Abcam	Cat#bb22758
Rabbit anti-pKAP	Bethyl Laboratories	Cat#A300-767A
Mouse anti-actin	Sigma	Cat#A5441
Mouse anti-CHK2	Upstate	Cat#05-649
Rabbit anti-PCNA	Merck	Cat# 07-2162
Rabbit anti SETD8	Sigma	Cat# HPA064495
Mouse anti-H2A	Cell Signaling	Cat#3636
Mouse anti-Tubulin	Sigma	Cat# T9026
Rabbit anti-H4K20me1	Abcam	Cat#9051
Rabbit anti-H4K20me1	Upstate	Cat#07-440
Rabbit anti-p21	Cell Signaling	Cat#2947
Anti-Mouse IgG-555	Bethyl Laboratories	Cat#A90-516D3
Anti-Mouse IgG-488	Invitrogen	Cat#A11001
Anti-Rabbit IgG-488	Invitrogen	Cat#A21441
Anti- Rabbit IgG-555	Bethyl Laboratories	Cat#A120-201D3
Anti- Rabbit IgG-647	Invitrogen	Cat#A21443
Anti-Rat IgG-488	Invitrogen	Cat#A21470
Mouse anti-IgA-RPE	Oxford Biotechnology	Cat#104009
Rat anti-HA	Merck	Cat#11867423001
Chemicals, peptides, and recombinant proteins		
Anti-CD40	BD Pharmingen	553722
IL-4	Preprotec	214-14
IL-3	Preprotec	213-13
CellTiter-Glo	Promega	G7571
Rapamycin	Alpha Aesar	J62473
TGF-β	R&D Systems	240-B
UNC0379	Selleckchem	S7570
HU	Sigma	H8627
4-OHT	Sigma	H7904
Critical commercial assays		
Click-It EU 488 Imaging kit	Thermo Fisher scientific	C10329
Gentra Puregene Blood Kit	Qiagen	Cat#158445
RnaiMax	Thermo Fisher scientific	Cat#13778150
Lipofectamine 2000	Invitrogen	Cat#11668027
QuantSeq 3' mRNA-Seq Library Prep Kit	Lexogen	N/A
Deposited data		
TPP proteomics data	PRIDE repository	PXD051199
Experimental models: Cell lines		
U2OS	ATCC	RRID:CVCL_0042
MCF7	ATCC	RRID:CVCL_0031
KBM7	Gift from T. Brummelkamp	RRID:CVCL_A426
SH-SY5Y	ATCC	RRID:CVCL_0019

TABLE S2. List of reagents used in this study.

NGP	Gift ATCC	RRID:CVCL_2141
P53$^{+/+}$ MEF	This paper	N/A
P53$^{-/-}$ MEF	This paper	N/A
Ba/F3_MycER	Gift Bruno Amati	Donati et al. 2022
FL5.12_MycER	Gift Bruno Amati	Donati et al. 2022
Setd8 +/- mESR	Gift Danny Reinberg	Oda et al. 2009
Setd8 lox/- mESR	Gift Danny Reinberg	Oda et al. 2009
Oligonucleotides		
Setd8_sg1 ACGGAGCGCCATGAAGTCCG	This paper	N/A
Setd8_sg2 ACGGGAGGCTCTGTACGCAC	This paper	N/A
Recombinant DNA		
pLEX305_SETD8_dTAG	Addgene	Addgene (Backbone #91798)
p-EF1a-CreERT2-3Xflag-T2A-eBFP2	Addgene	Addgene (Plasmid #170186)
LentiCas9_Blasti	Addgene	Addgene (Plasmid 52962)
pLenti CRISPR.V2_ Blasti_sg1SetD8	This paper	Addgene (Backbone #98293)
pLenti CRISPR.V2_Blasti_sg2SetD8	This paper	Addgene (Backbone #98293)
Software and algorithms		
GraphPad Prism	GraphPad Software Inc	http://www.graphpad.com/scientific-software/prism/
